# Social identity, social networks and recovery capital in emerging adulthood: A pilot study

**DOI:** 10.1186/s13011-015-0041-2

**Published:** 2015-11-11

**Authors:** E. Mawson, D. Best, M. Beckwith, G. A. Dingle, D. I. Lubman

**Affiliations:** Eastern Health Clinical School, Monash University, Melbourne, Australia; Turning Point, Melbourne, Australia; Department of Law and Criminology, Sheffield Hallam University, Sheffield, S10 2BP UK; School of Psychology, University of Queensland, Brisbane, Australia; Centre for Youth Substance Abuse Research, University of Queensland, Brisbane, Australia

**Keywords:** Social network, Social identity, Emerging adult, Substance use, Treatment, Recovery, Quality of life

## Abstract

**Background:**

It has been argued that recovery from substance dependence relies on a change in identity, with past research focused on ‘personal identity’. This study assessed support for a social identity model of recovery in emerging adults through examining associations between social identity, social networks, recovery capital, and quality of life.

**Methods:**

Twenty participants aged 18–21 in residential treatment for substance misuse were recruited from four specialist youth drug treatment services - three detoxification facilities and one psychosocial rehabilitation facility in Victoria, Australia. Participants completed a detailed social network interview exploring the substance use of groups in their social networks and measures of quality of life, recovery capital, and social identity.

**Results:**

Lower group substance use was associated with higher recovery capital, stronger identification with non-using groups, and greater importance of non-using groups in the social network. Additionally, greater identification with and importance of non-using groups were associated with better environmental quality of life, whereas greater importance conferred on using groups was associated with reduced environmental quality of life.

**Conclusions:**

Support was found for the role of social identity processes in reported recovery capital and quality of life. Future research in larger, longitudinal samples is required to improve understanding of social identity processes during treatment and early recovery and its relationship to recovery stability.

## Background

Emerging adulthood, from 18 to 25 years of age, forms a distinct developmental period marked by identity uncertainty and exploration as young people experiment with the various identities available to them within their personal and social contexts [1]. Emerging adulthood is consistently associated with higher risk for onset of psychological disorders, problematic substance use and onset of substance use disorders [2, 3]. Up to 13 % of those aged 16–24 have been found to engage in problematic substance use in Australia [2]. Indeed, the majority of adults with substance use disorders report early onset of use, with early onset associated with lifetime consequences including poorer health, wellbeing, and social functioning. The Victorian Youth Cohort Study found that amongst treatment seekers aged 16–21 years, the majority engaged in ongoing or repeated treatment experience across multiple service providers, including specialist Alcohol and/or Other Drug (AOD), mental health, and housing support services [4].

In this context, this pilot study sought to explore the extent to which emerging adults’ personal and social resources for recovery, or recovery capital [5–7], while in residential treatment for alcohol and/or drug use disorders was associated with the substance use of groups within their social networks, with the aim of highlighting the importance of social networks external to the treatment setting to treatment planning and provision.

### Recovery capital, social networks and identity

Recovery capital refers to “the resources that can be brought to bear on the initiation and maintenance of substance misuse cessation” (p.1972) [5]. Conceptually, recovery capital can be present whilst in the midst of active addiction, contributing to an individual’s motivation and capacity to initiate treatment, life satisfaction, and maintenance of recovery following treatment. Social recovery capital refers to the opportunities and benefits associated with social group memberships and family relationships supportive of recovery [8], and includes access to material, informational, and emotional social supports, prosocial drive and reciprocity, and social expectancies that may support motivation when faced with personal challenges to recovery. Personal recovery capital refers to the resources and skills the person possesses that promote or limit their capacity for recovery, including material resources, education, physical and psychological health, coping and problem solving skills, sense of meaning and purpose, and self-efficacy for recovery [8]. Although differing resources, personal and social capital evolve in a dynamic relationship and are increased or depleted over time as the effects of substance misuse – or recovery – impact on the individuals’ personal and social functioning.

Research into social network effects on treatment suggests that high social recovery capital may moderate the effects of reduced personal recovery capital [5]. In clinical populations, decreased support for substance use and increased support for sobriety within the social network has been associated with reduced risk for relapse to alcohol use [9–12], and drug use [13]. Post-treatment outcomes were most strongly predicted by the number of people in the network, the proportion of the pre-treatment network abstinent or in recovery [14] and addition of friends who were abstinent or in recovery [14]. In the general population, there is evidence for the spread of substance use behaviours across social networks, with individual alcohol consumption higher in social networks where more members drank heavily, and decreasing in social networks where more members abstained^1^ [15].

Despite the association between individual and social network substance use behaviours, few studies have explored psychological mechanisms that link social networks to treatment outcomes. Instead, existing studies have focused on how social groups exert an external influence on behaviour via social learning, reciprocity, and mutual obligation [16–18]. In line with recent research [19], we propose that Social Identity Theory [20] provides a model for how the social capital afforded by the social network translates to individual treatment experiences and the development of a non-using social identity [21]. Social Identity Theory proposes that individuals adopt the normative values and behaviours of groups they belong to (i.e. their in-group), while minimising the salience of values and behaviours of groups they do not feel they belong to (the outgroup). The subjective sense of belonging to a group and the importance of that group membership to the individual’s sense of self are key to understanding how groups guide behaviour [22]. In the context of recovery, a social identity approach focuses on substance use related values and behaviours that characterise groups in the social network, the importance of groups low in substance use, and the subjective perception of incongruence of heavy substance using groups with the recovery goals of the individual.

Within this model, it is membership of groups whose normative attitudes and behaviours to substance use are congruent with the individual’s recovery goals that enables access to social capital [22]. Social identity change is reflected in a poor fit between earlier substance-using groups and the new recovery goals [23, 24], prioritising of recovery-congruent group memberships over groups engaged in substance use, and distancing from using groups in the social network [25]. Paralleling cultural-identity models in which identification with drug using groups supports the emergence of a drug using identity [26], access to recovery-congruent groups in the social network appears to support and grow in tandem with change toward a recovery identity [25].

In support of the position that social identity processes may be present in recovery, the strength of identification with a social group and the groups' smoking-related norms best accounted for the association between group smoking and individuals’ future smoking in emerging adults aged 17–20 [27]. In a younger cohort of adolescents aged 14–18 [28], the effectiveness of support for AOD treatment provided by a low substance-using social network increased with stronger identification with that network, whilst risk for relapse increased amongst those who strongly identified with social networks characterised by high levels of substance use. However, poorer outcomes associated with high substance-use within these social networks were ameliorated when identification with the network was low.

Amongst older adults, moderate identification with substance-using social groups, and high identification with a Therapeutic Community (TC) following community entry predicted treatment retention and completion [29]. Controlling for differences in substance use severity and TC identification at treatment entry, a greater transition from a “user” to “recovery” social identity accounted for substantial variance in drinking quantity (34 %), drinking frequency (41 %), and life satisfaction (49 %) at treatment completion [30]. Finally, higher preference for recovery groups in the general community (e.g., Alcoholics Anonymous) over using groups in the social network was associated with higher recovery self-efficacy and more months substance-free [19].

That social identity change during treatment may be associated with treatment outcomes is relevant to early interventions in emerging adult populations that aim to prevent onset of long-term substance using careers in adulthood. However, no studies have explored associations between social identification with using or non-using groups and recovery capital in emerging adulthood. This gap in the literature is a concern, as it cannot be assumed that prior associations between social networks, treatment experiences and recovery capital generalise to the specific developmental demands and challenges of emerging adulthood. The aim of this pilot study was to assess the degree to which social group substance use, and identification with using or non-using groups in the social network, is associated with personal and social recovery capital and quality of life in emerging adults in residential AOD treatment. The second aim is to identify opportunities for ongoing support for emerging adults with substance use problems in relation to their social networks, social identities, recovery capital and quality of life. To this end, three hypotheses were tested:

1. Lower substance use of groups in the individual’s social network are associated with higher recovery capital and quality of life.
2. Higher identification with non-using groups in the individual’s social network are associated with higher recovery capital and quality of life.
3. Higher identification with groups engaged in problematic substance use are associated with lower recovery capital and quality of life.

## Method

### Participants

Participants were 15 males and 5 females (mean age = 19.8, *SD* = 0.8), recruited from three youth residential detoxification facilities located in inner Melbourne (*n* = 10), suburban Melbourne (*n* = 1), regional Victoria (*n* = 4), and one residential rehabilitation facility (*n* = 5) in outer Melbourne, with all facilities operated by a single youth outreach and treatment agency focused on service provision to vulnerable youth. Inclusion criteria included current residence in a publically funded youth AOD treatment facility and age 18–21 years. Exclusion criteria included active psychosis or significant emotional or cognitive impairment as determined by clinical staff at each facility.

Eligibility for admission and current residence in the treatment facility were taken as proof of prior alcohol and/or drug use disorder warranting clinical intervention. Due to time constraints the recruitment period was limited to a four-week period, and the the final cohort is a convenience sample of all the eligible participants at each treatment facility who consented to participate. A majority of eligible residents at each facility chose participation, suggesting that the sample was representative of 18–21 year old residents of publicly funded AOD residential treatment facilities in Victoria, Australia.

## Materials

### Demographics questionnaire

Data were collected on age, gender, past 28-day employment and/or education, usual accommodation, current treatment duration, and prior treatment experience.

### World Health Organisation Alcohol, Smoking, and Substance Involvement Screening Test (WHO ASSIST 3.0) [31]

Question two of the ASSIST 3.0 assesses past 28-day frequency of use for a range of substances on a five-point Likert scale (0 = Never, 4 = Daily or almost daily). The ASSIST subscale has demonstrated concurrent validity to the Addiction Severity Index (*r* = 0.71–0.89.

### Assessment of Recovery Capital scale (ARC) [32]

Assessment of Recovery Capital (ARC) is a 50-item self-report measure of two domains of recovery capital, personal recovery capital, and social recovery capital. Items are dichotomously scored (0 = no, 1 = yes) providing for a score range of 0–25 within each domain, with higher scores indicating higher capital. The ARC generates a single factor with strong inter-class correlation coefficients (ICC = 0.50–0.73) between items [32]. The internal consistency of the ARC total, personal, and social domain scale scores assessed with Cronbach’s alpha coefficients have not been previously reported, and were good in the current study (α = .89–96).

### World Health Organisation Quality of Life – BREF (WHOQOL-BREF) [33]

The WHOQOL-BREF is a 26-item self-report measure of subjective Quality of Life (QOL) in social, environmental, psychological and physical health domains. Item responses are rated on a five-point Likert-type scale (1 = Not at all, 5 = Completely). WHOQOL-BREF domains have previously demonstrated acceptable internal consistency (α = 0.66–0.84) and concurrent validity with the WHOQOL-100 [33].

### Social identity map

The social identity map is a graphic representation of respondents’ social networks, and yields quantitative data regarding (i) number of groups in the network; (ii) group importance (very, moderately, somewhat); and (iii) substance use status of the members of each group, based on the conceptual model outlined by Jetten and colleagues [34, 35].

Perceived level of group substance use documented in the maps is a self-report measure, and follows conventions established in Project MATCH for assessing severity of alcohol consumption in the social network from abstinent to light drinker, moderate drinker, or heavy drinker [9]. Participants rated the substance use of individual group members as non-use/abstinent, non-problematic (e.g., light-moderate) use, or problematic (e.g., heavy) use. Group substance use categories were based on the most frequent category assigned to the members of that group, with non-use coded as 0, non-problematic use coded as 1, and problematic use as 2.

## Exeter Identity Transition Scales (ExITS) [36]

### Past and current group memberships

The ExIT scales assess the number, importance, congruence, and strength of identification with up to six groups on seven-point Likert-style scales. The current study only assessed strength of group identification and asked respondents to indicate their agreement with the statement “I identify with [ingroup name]” (1 = disagree completely, 7 = agree completely). Prior research has found these scales provide a valid measure of the range and quality of group memberships held [29, 36, 37].

### Procedure

Study approval was granted by the Monash University Human Research Ethics Committee and Eastern Health Human Research Ethics Committee. Recruitment was conducted via recruitment flyers that asked “Are you interested in being part of a study exploring identity and social networks of emerging adults in treatment for alcohol and/or other drug problems?”, followed by a description of the type of questions to be asked, time needed and payment for participation, and a statement that a decision to participate was restricted to those aged 18 or more and voluntary.

Informed consent preceded all interviews and emphasised that participation, or withdrawal from participation, would not influence ongoing treatment. Interviews ranged from 50–70 min duration and participants were paid $20 for their involvement. This value is lower than that reported in other youth substance use research in Australia [38], and is considered large enough to act as a mark of appreciation without inducing participation motived only by financial gain. Supporting this, several participants stated that their decision to participate had been motivated by the opportunity to be heard.

### Study design and analysis

The study employed a correlational, cross-sectional design. All analyses were conducted in SPSS Statistics 21.0 and assessed at the conventional significance level of *p* < .05. Power analyses conducted in G*Power, based on calculations for a Type 1 alpha level of .05 and a Type II error rate of .80, suggested that the study was underpowered to detect all but large effects above *r*^*2*^ = .30.

Descriptive statistics were undertaken for demographic, substance use, recovery capital, quality of life, and social network measures. Comparative data was included in descriptive statistics tables to contextualize the current results, with larger differences between groups tested for significance. Independent groups t-tests were conducted with published group means, standard deviations, and participant numbers using syntax published by Field [39]. Cohen’s *d* was calculated for significant results to indicate the magnitude of the difference using an online calculator (http://www.danielsoper.com/statcalc3), with small (<0.2), medium (0.2–0.8) and large (>0.8) effect sizes evaluated in line with the recommendations of Gravetter and Wallnau [40].

Zero-order Pearson’s correlations were conducted between personal and social recovery capital, quality of life, group substance use on a continuum from non-using/abstinent (0) to heavy use (2), group importance, and group identification. Individual differences in these domains were not statistically controlled, preserving external validity in the context of treatment populations characterised by high heterogeneity. When significant, *r*^2^ effect sizes were computed to indicate the magnitude of the effect as an indicator of potential clinical significance, with weak (<.09), medium (0.09–0.25) and large (>.25) effects evaluated in line with the recommendations of Gravetter and Wallnau [40]. Finally, 95 % Confidence Intervals [CI] were calculated to indicate the probable range within which the true correlation may be predicted to fall and are reported in text.

## Results

### Demographic information

#### Employment, education and accommodation

Participants included 15 males and 5 females with a mean age of 19.8 years (*SD* = 0.8). No participants reported current employment. One participant reported engaging in 16 days of education and training, and three reported 1–4 days of education associated with their treatment program in the past 28 days. Ten participants reported living with their family prior to treatment entry. For those who indicated usual residence outside of the family home, three reported private rental, one reported “staying with friends”, two reported “own home”, two reported being homeless, one reported transitional public housing accommodation, and one reported boarding house accommodation prior to treatment entry.

### Substance use, treatment experience and wellbeing

Participants reported a mean duration of current treatment of 19 days (*SD* = 28.2) and median treatment length of 4.5 days, with most of the sample recruited from short-term detoxification facilities. Fourteen participants reported at least one prior substance use treatment including AOD counseling (*n* = 8), residential withdrawal and/or rehabilitation (*n* = 5), and opiate substitution therapy (*n* = 1). The most frequently used substances were alcohol, cannabis, and amphetamines.

### Social networks

All participants reported belonging to at least one social group, 19 reported at least 2–3 groups, 15 reported four or more groups, nine reported five or more groups, and three participants reported six group memberships. Social network groups typically consisted of immediate family (23.5 % of all groups identified, *n* = 20), extended family (10.6 %, *n* = 9), partners (9.4 %, *n* = 8), friends (35.3 %, *n* = 30), substance-using friends (10.6 %, *n* = 9), support services (7.1 %, *n* = 6), and recovery friends (3.5 %, *n* = 3)^2^. A small increase was reported in social network size from pre-treatment, with three participants recruited from long-term psychosocial rehabilitation including their recovery community as part of their current social network.

**Fig. 1:**
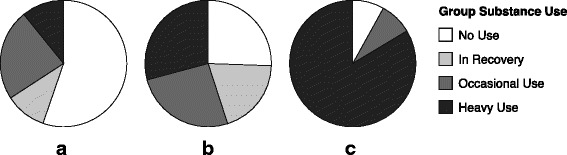
Substance use status of groups rated **a** very important, **b** moderately important, **c** somewhat important

Figure 1 presents the proportions of non-using, in recovery, occasional-using, and heavy-using groups at each level of group importance. Of the groups rated as very important (n = 47), non-using groups formed a significantly larger proportion (55.3 %, *n* = 26), than in recovery groups (10.6 %, *n* = 5), occasional (23.4 %, *n* = 11) or heavy (10.6 %, *n* = 5) substance-using groups, χ ^2^ (3, *n* = 46) = 23.22, *p* = .000. Participants also reported significantly higher identification with groups in their networks that were abstinent or in recovery (*M* = 2.34, *SD* = 1.68), than with groups engaged in heavy substance use (*M* = 1.19, *SD* = 0.93), *t*(19) = 3.07, *p* = .006, *d* = 0.69.

### Recovery capital and quality of life

Mean scores for total, personal and social recovery capital and facets of each domain are reported in Table 1, alongside previously published normative scores for older individuals recruited in the United Kingdom. As can be seen in Table 1, mean total and domain scores appear to be in-line with normative scores in adult populations published by Groshkova, Best and White [32] for a mixed treatment and post-treatment recovery sample, however are lower than those reported by Best, Honor et. al., [41] in an adult recovery only (e.g., non-treatment) sample. As seen in Table 1, emerging adults showed lower personal, social and total recovery capital than in previous research. Independent samples t-tests found significant differences between participants in the current study and older adults in recovery reported by Best and colleagues [41] for social (*t*(194) = −5.97, *p* = .0001, *d* = 1.26), personal (*t*(194) = −6.33, *p* = .0001, *d* = 1.13), and total recovery capital (*t*(194) = −6.06, *p* = .0001, *d* = 1.22).

**Table 1:** Mean total, domain and facet scores on the assessment of recovery capital scale

Variable	Current Study Mean (SD)	Possible Range	Mixed Treatment and Recovery Mean (SD)^a^	Recovery Only Mean (SD)^b^
Social	14.30 (5.71)	0–25	14.63 (−)^c^	20.70 (4.40)^*^
Substance Use & Sobriety	2.50 (1.50)	0–5	2.58 (1.43)	*NR*
Community Involvement	2.80 (1.32)	0–5	3.10 (1.70)	*NR*
Social Support	3.05 (1.70)	0–5	2.93 (1.67)	*NR*
Housing and Safety	3.15 (1.42)	0–5	2.87 (1.59)	*NR*
Meaningful Activities	2.80 (1.54)	0–5	3.15 (1.47)	*NR*
Personal	15.80 (6.10)	0–25	16.62 (−)^c^	21.40 (3.40)^*^
Psychological Health	3.00 (1.30)	0–5	3.44 (1.38)	*NR*
Physical Health	3.10 (1.77)	0–5	3.24 (1.60)	*NR*
Risk Taking	3.15 (1.27)	0–5	2.98 (1.33)	*NR*
Coping and Functioning	2.75 (2.05)	0–5	3.31 (1.58)	*NR*
Recovery Experience	3.80 (1.15)	0–5	3.65 (1.63)	*NR*
Total	30.10 (11.42)	0–50	31.25 (11.54)	42.10 (8.00)^*^

*Note.* Significant differences to current results at *p < .001 *NR* = Not Reported. ^a^Mixed treatment and recovery sample (*n* = 144), mean ages 35.1 (±12.3, treatment sample) and 41.5 years (±9.1, recovery sample), reported from Groshkova, T., Best, D., & White, W. (2012). The Assessment of Recovery Capital: Properties and psychometrics of a measure of addiction recovery strengths. *Drug and Alcohol Review*. ^b^Recovery-only sample (*n* = 176), mean age 41.5 (±9.1) years, reported from Best, D., Honor, S., Karpusheff, J., Loudon, L., Hall, R., Groshkova, T., & White, W. (2012). Well-being and recovery functioning among substance users engaged in posttreatment recovery support groups. *Alcoholism Treatment Quarterly*, *30*(4),397-406. ^c^Not reported but calculated through summing the facets of each domain

Table 2 presents mean Quality of Life (QOL) domain scores on the WHOQOL-Bref alongside normative scores for a sample recruited in longer recovery periods (up to 5 years) and for near-age peers in the general population. As seen in Table 2, emerging adults in the current study showed lower QOL across all domains than older adults with more time spent in recovery [42], who in turn showed lower QOL compared to the general population [43]. Independent samples t-tests found significant differences between emerging adult participants, adult recovery groups, and general population norms for psychological (*t*_*older*_(53) = −4.22, *p* = .0001, *d* = 1.13; *t*_*norm*_(59) = −5.02, *p* = .0001, *d* = 1.35), physical (*t*_*older*_(53) = −2.31, *p* = .03, *d* = 0.63; *t*_*norm*_(59) = −6.16, *p* = .0001, *d* = 1.51), and environmental QOL (*t*_*older*_(53) = −2.17, *p* = .05, *d* = 0.58; *t*_*norm*_(59) = −3.83, *p* = .0003, *d* = 0.92). Social QOL significantly differed between current participants and the general population (*t*_*norm*_(59) = −3.83, *p* = .0003, *d* = 0.99), but did not significantly differ between current participants and the older recovery group.

**Table 2:** Mean domain scores on the World Health Organisation quality of life-brief scale

Domain	Current Study Mean (±SD)	Possible Range	Early Recovery^a^ Mean (±SD)	Normative Data^b^ Mean (±SD)
Psychological	46.79 (18.89)	0–100	65.24 (13.44)^**^	71.4 (17.5)^**^
Physical	61.25 (19.78)	0–100	72.92 (17.01)^*^	85.4 (10.9)^**^
Social relations	51.16 (24.56)	0–100	62.26 (20.44)^†^	72.9 (18.8)^**^
Environment	57.81 (21.18)	0–100	68.70 (15.81)^*^	74.3 (14.0)^**^

*Note.* Significant differences to current results: †*p* < .10 (trend) *p < .05. **p < .001. ^a^Recovery defined as recovery duration < 5 years, *n* = 35, mean age = 42.6, from Hibbert, L. J., & Best, D. W., (2011). Assessing recovery and functioning in former problem drinkers at different stages of their recovery journeys. *Drug and Alcohol Review*, 30,12–20. ^b^General population norms in Victoria, Australia, *n* = 41, age range = 20–29, from Hawthorne, G., Herrman, H., & Murphy, B. (2006). Interpreting the WHOQOL-BREF: Preliminary norms and effect sizes. *Social Indicators Research, 77*, 37–59

### Associations between social network groups, recovery capital, and quality of life group substance use, group identification and importance

Table 3 presents the matrix of correlations for group substance use, personal and social recovery capital, identification with groups, importance of groups, and quality of life. Higher substance use by groups in the social network was significantly associated with higher identification with heavy-using groups (95 % CI [.31, .85], *r*^*2*^ = .44), higher importance of heavy-using groups (95 % CI [.32, .86], *r*^*2*^ = .45), lower identification with non-using groups (95 % CI [.57, .92], *r*^*2*^ = .66) and lower importance of non-using groups (95 % CI [.71, .95], *r*^*2*^ = .77). Finally, higher importance of heavy-using groups was significantly associated with lower importance of non-using groups (95 % CI [−.75, −.03], *r*^*2*^ = .22).

**Table 3:** Correlations between recovery capital, quality of life, and social network substance use, identification, and importance

	ARC		WHOQOL-BREF				Social Network			
Variable	Personal	Social	Physical	Psych	Social	Enviro	Group substance use	Non-using groups identification	Heavy using groups identification	Non-using groups importance
ARC										
Personal	–									
Social	.87^***^	–								
WHOQOL-BREF										
Physical	.74^***^	.54^**^	–							
Psychological	.62^**^	.52^*^	.61**	–						
Social	.39^†^	.52^*^	.24	.68^***^	–					
Environmental	.58^**^	.72^***^	.49^*^	.46^*^	.41^†^	–				
Social Network										
Groups substance use status	-.50^*^	-.47^*^	-.39	-.10	.02	-.44^†^	–			
Non-using groups identification	.37	.44^†^	.24	.03	.05	.53^*^	-.81^***^	–		
Heavy-using groups identification	-.32	-.29	-.25	-.08	-.14	-.38	.66^**^	-.41	–	
Non-using groups importance	.33	.38^†^	.26	-.07	-.08	.48^*^	-.88^***^	.94^***^	-.44^†^	–
Heavy-using groups importance	-.43^†^	-.39^†^	-.38^†^	-.10	-.08	-.47^*^	.67^**^	-.42^†^	.90^***^	-.47^*^

Note. *ARC* Assessment of Recovery Capital, *WHOQOL-BREF* World Health Organisation Quality of Life-Bref

†*p* < .10 (trend) *p < .05. **p < .01. ***p < .001

### Recovery capital and quality of life

Greater substance use by groups was significantly associated with lower personal recovery capital (95 % CI [.07, .77], *r*^*2*^ = .25) and social recovery capital (95 % CI [.04, .76], *r*^*2*^ = .22). All QOL domains were significantly associated with social recovery capital, however environmental QOL showed the strongest association (95 % CI [.41, .88], *r*^*2*^ = .52). Significant associations were found between higher environmental QOL and higher identification with non-using groups (95 % CI [.11, .79], *r*^*2*^ = .28), higher importance of non-using groups (95 % CI [.05, .76], *r*^*2*^ = .23), and lower importance of heavy-using groups (95 % CI [.03, .76], *r*^*2*^ = .22). A significant association was also found between psychological QOL and social QOL (95 % CI [.34, .86], *n* = 20, *r*^*2*^ = .46).

## Discussion

The aim of this pilot study was to assess the degree to which substance use in social networks, and identification with using or non-using groups, was associated with recovery capital and quality of life in emerging adults in residential AOD treatment, with a second aim of identifying opportunities for ongoing support for emerging adults with substance use problems in relation to their social networks, social identities, recovery capital and quality of life.

H1: The first hypothesis, that lower substance use among groups in the social network would be associated with higher recovery capital and quality of life, was partially supported with lower group substance use associated with higher recovery capital, but not quality of life. The finding that group substance use is associated with recovery capital is consistent with prior findings that lower social network support for substance use predicted reduced substance use and relapse at follow-up in adult [9–13] and adolescent populations [28], and extends this finding to a treatment population in the bridging period of emerging adulthood. The associated medium to large effect sizes for recovery capital suggest that identification with groups that engage in lower levels of substance use may be of clinical relevance for the accrual of recovery resources that can be drawn on to sustain treatment motivation and support post-treatment outcomes.

H2: The second hypotheses that greater identification with non-using groups in the social network would be associated with higher recovery capital and quality of life was supported only for environmental QOL, which showed associations to higher identification with and importance of non-using groups and reduced importance of heavy using groups. Considered in conjunction with the finding that over half the variance in social recovery capital was accounted for by environmental QOL, it appears that factors that contribute to environmental QOL, including access to safe accommodation, safe environment, income, transport, services, information, and leisure opportunities [44], are fundamental aspects of social and community capital that supports recovery.

In addition, it is tentatively noted that there were trends toward significance for associations between social recovery capital and higher identification with and importance of non-using groups. Although this may not necessarily be an issue of statistical power, it may suggest that social identity factors are associated with the perceived relevance, acceptability and utility of social resources for achieving recovery goals. If so, the relative availability of non-using groups compared to heavy-using groups in the social network may influence whether the recovery resources that non-using groups provide are perceived to be important and congruent with one’s personal recovery goals and identity. This prediction would be in line with the findings of Vik and colleagues [28] suggesting that the perceived utility of social support in adolescent AOD treatment varied according to identification with the sources of support. Future studies to examine the perceived relevance of supports and resources associated with both heavy-using and non-using groups in the lived environment may shed additional light on processes that link social identity, recovery capital, and environmental quality of life as experienced outside of residential treatment.

H3: The third hypothesis, that higher identification with heavy-using groups would be associated with lower recovery capital and quality of life, was not supported. This suggests that gains in wellbeing stemming from higher identification with non-using groups represents the emergence of social resources that exists in parallel with the reductions to wellbeing associated with ongoing identification with using groups, rather than simply a reversal of lower wellbeing associated with identification with high substance using social groups.

The lack of association between identification with heavy-using groups, recovery capital and quality of life was unexpected, but consistent with prior studies that reported no *direct* associations between social identity and substance use at follow-up [19]. Indeed, Buckingham and colleagues [19] found that greater *preference* for non-using groups in the social network predicted higher recovery self-efficacy and more time substance free, and in line with this we observed that the *importance* attached to high using groups showed a near significant association to lower social recovery capital. Finally, we observed high multi-collinearity of social identification and importance, and it appears that *identification*, *preference*, and *importance* of social groups are closely related constructs that are not yet clearly differentiated, but that each support increased psychological accessibility of social resources and learning that support recovery.

### Other findings

In spite of mixed support for the social identity hypotheses, non-using groups comprised a larger proportion of “very important” groups in the networks of emerging adults in AOD treatment, with emerging adults reporting significantly higher identification with non-using than with heavy-using groups overall. These are novel findings in the literature on AOD treatment and recovery in emerging adult populations, but in accord with prior research in older recovery populations in an Australian therapeutic community [29]. Together, these studies suggest that differences in the relative importance of non-using and heavy-using groups are evident in both early and later treatment stages, and in treatment populations at differing age-related developmental stages.

Comparisons of recovery capital showed that emerging adults in early treatment showed significantly lower personal, social and total recovery capital compared to older adults in recovery for up to five years [41]. This is in line with literature suggesting that personal and social resources continue to strengthen and grow with time in recovery as the new recovery identity and lifestyle grows [42]. In the context of no significant differences in recovery capital reported by a mixed recovery and treatment sample of older adults, this also suggests that gains in recovery capital do not differ as a function of mean age in treatment.

Similarly, comparisons of quality of life suggest that emerging adults experience specific challenges in psychological wellbeing, physical health, relationships, and daily living conditions compared to both older adults in recovery and to same aged peers in the general community, with particularly large effects for the gap in quality of life compared to same aged peers. Finally, no significant differences between emerging adults and older adults in recovery in their quality of social relations suggests that both groups experience significantly lower social QOL compared to the general population, indicative of specific and potentially persistent challenges in social connectedness reported by those in AOD recovery.

### Implications for AOD policy and treatment

Due to the small sample size recruited in this study, the following recommendations must be regarded as tentative and to be treated with caution until our findings are corroborated in future studies. However, with this caveat, several implications for the provision of AOD treatment in emerging adult populations are noted. These findings argue for the importance of treatment strategies that consider the role of social networks in treatment outcomes, both for relapse, and for potentially protective effects of increased social capital associated with low-using groups. Through social identification, social networks are not only an external entity outside of treatment, but form an active ingredient brought by individuals into the treatment setting. Across previous studies, this internalized social identity has been associated with a range of treatment and post-treatment factors captured by the construct of recovery capital, including recovery self-efficacy, coping [19], and expectancies regarding the utility and sustainability of recovery outside of treatment [28]. As such, consideration should be given to the development and evaluation of interventions that encourage emerging adults to review and reflect on the importance of heavy-using and non-using groups and to increase engagement with non-using groups in their social networks.

Following from this, a number of avenues for assertive linkage to pro-social groups and activies are suggested, reflecting existing literature that acknowledges social and structural differences in the distribution of social recovery capital across communities [8, 45]. Targeted efforts to restore and/or maintain conditions necessary for improved environmental QOL following residential treatment, such as accommodation support, supported linkage to employment and training opportunities, and opportunities for leisure activities congruent with recovery goals, may directly contribute to the creation of social recovery capital. Such support for increased environmental quality of life may influence the degree to which non-using group’s experiences, opportunities, and values are perceived of as similar to the experiences, opportunities and values of the self, laying the foundation for a sense of shared understanding and group belonging that is the core of social identity [22]. In turn, greater identification with and importance of non-using groups should predict the degree to which non-using groups will be drawn on in preference to heavy-using groups to support wellbeing and recovery when faced with challenges following treatment [19, 22, 24, 25].

Finally, assertive linkage to groups that engage in low levels of substance use may be especially important for emerging adults whose pre-treatment social networks engage in heavier substance use, and who continue to strongly identify with heavy-using groups at treatment completion [5]. In this context a key aim of assertive linkage is to support continued connectedness to non-using groups in the social environment, keeping the door open for incremental gains in social recovery capital that supports increased recovery capacity over time. At a practical level, such assertive linkage supports social recovery capital, environmental quality of life, ongoing opportunities to learn from recovery role models, and participation in social activities that provide social connectedness congruent with recovery [5, 18].

### Limitations

Several important limitations are noted. As a pilot project assessing the feasibility of the social identity mapping model with youth populations, the small sample size suggests that some non-significant effects may reflect limited statistical power, and the findings and conclusions should be interpreted with caution. Limited sample size also precluded the feasibility of more sophisticated meditational analyses of social identity processes on recovery capital, whist the cross-sectional design prevented assessment of causal pathways for change in identification with using and non-using groups over time in treatment or on post-treatment outcomes. The small sample size may also have resulted in unstable correlations [46]. However, the lower bound of the 95 % confidence intervals reported for key associations between group identification, importance, group substance use and environmental quality of life suggest that these associations would be likely to remain significant across the range of values within which the true score may be expected to fall.

The focus on emerging adults with poly-substance use histories and in residential treatment may limit the degree to which these findings generalise to populations differing in age, substance use profile, or treatment modality. Furthermore, social networks may be of greater importance in emerging adulthood, and emerging adults likely have greater contact with and place greater importance on peer groups than at later stages of the lifespan. However, whilst this may have magnified the strength of associations in the current study, it is of note that quality of social relations did not differ to older adults in recovery but did differ to peers in the general community, suggesting that effects of substance misuse override age-related effects on quality of social relations. Finally, weighting of the sample toward participants in early detoxification may have attenuated the degree of change observed in social networks and identity.

This study was used as a pilot study to help refine the social mapping technique for a subsequent longitudinal study with adults in recovery in residential treatment, and as a pilot trialed the acceptability of a broad questionnaire examining recovery identity and wellbeing, and feasibility of social identity mapping as a technique for capturing the substance use of groups in the social network. Future research utilising a mixed-methods longitudinal design in a larger sample and recruited across a range of treatment modalities and ages is required, and a larger treatment outcome study is now examining the impacts of social identity transition on recovery wellbeing using a mixed-methods longitudinal design in a sample of adults accessing residential treatment for alcohol and drug problems.

Key next steps include; (i) Replication and extension of current findings using a longitudinal design in a larger sample recruited across a range of treatment modalities; (ii) inclusion of a control group representing the substance use of groups in the social networks of individuals in nonclinical settings; (iii) Evaluation of interventions encouraging individuals in AOD treatment to reflect on the ways that groups in their social network contribute to their sense of identity, coping and recovery resources following treatment; (iv) Evaluation of the predictive importance of social networks on clinical and wellbeing outcomes (v) Exploration of the utility of interventions to increase the salience of non-using groups in the social network, including evaluation of the feasibility and acceptability of linkage to non-using groups for emerging adults who report few non-using groups and identify strongly with using groups in their social networks, and; (v) Evaluation of associations between targeted after-care support to improve quality of life in daily living conditions and post-treatment recovery outcomes for emerging adult populations.

## Conclusion

This study examined associations between social network group substance use, group identification and importance, recovery capital, and quality of life in emerging adults in residential AOD treatment. Whilst the findings of this study should be regarded as provisional, four key findings extend current understanding of social identity processes in recovery in this population and point to future research directions. First, higher groups substance use was specifically associated with recovery capital but not quality of life, whereas identification with non-using groups was associated with environmental quality of life, but not with recovery capital or other quality of life domains. Second, environmental quality of life accounted for up to half of the variance in social recovery capital. Third, non-using groups were rated overall as more important than heavy-using groups, with group importance showing associations to recovery capital and quality of life. Fourth, lower recovery capital and quality of life in most domains suggest specific challenges experienced by emerging adults compared to older adults in recovery and to same-age peers in the general population. Our findings suggest that researchers and policy makers need to be more aware of the importance of social networks and their integral relationship to how young people see themselves, which has profound implications for treatment engagement and for adherence to treatment. Further research is needed to assess the predictive importance of social networks on treatment outcomes as a first step toward exploration of the utility and acceptability of interventions seeking to increase the importance of non-using groups in the social network.

## Abbreviations

AOD: alcohol and/or other drug
QOL: quality of life
TC: therapeutic community

## Footnotes

1 The authors would like to note treatment, and the term “non-user” or “non-using identity” is used to capture non-chaotic or non-problematic use and does not imply that abstinence is the only route to recovery

2 The labels for groups were provided by the participants and were not in response to pre-formed options driven by the researchers
